# Effect of Hypothermia in the Emergency Department on the Outcome of Trauma Patients: A Cross-Sectional Analysis

**DOI:** 10.3390/ijerph15081769

**Published:** 2018-08-17

**Authors:** Ting-Min Hsieh, Pao-Jen Kuo, Shiun-Yuan Hsu, Peng-Chen Chien, Hsiao-Yun Hsieh, Ching-Hua Hsieh

**Affiliations:** 1 Division of Trauma Surgery, Department of Surgery, Kaohsiung Chang Gung Memorial Hospital and Chang Gung University College of Medicine, 123 Ta Pei Road, Niao-Song District, Kaohsiung 833, Taiwan; hs168hs168@gmail.com; 2 Division of Plastic Surgery, Department of Surgery, Kaohsiung Chang Gung Memorial Hospital, Chang Gung University College of Medicine, 123 Ta Pei Road, Niao-Song District, Kaohsiung 833, Taiwan; bow110470@gmail.com (P.-J.K.); ah.lucy@hotmail.com (S.-Y.H.); VENU_CHIEN@hotmail.com (P.-C.C.); sylvia19870714@hotmail.com (H.-Y.H.)

**Keywords:** hypothermia, emergency department (ED), mortality, coagulopathy

## Abstract

This study aimed to assess whether hypothermia is an independent predictor of mortality in trauma patients in the condition of defining hypothermia as body temperatures of <36 °C. Data of all hospitalized adult trauma patients recorded in the Trauma Registry System at a level I trauma center between 1 January 2009 and 12 December 2015 were retrospectively reviewed. A multivariate logistic regression analysis was performed in order to identify factors related to mortality. In addition, hypothermia and normothermia were defined as temperatures <36 °C and from 36 °C to 38 °C, respectively. Propensity score-matched study groups of hypothermia and normothermia patients in a 1:1 ratio were grouped for mortality assessment after adjusting for potential confounders such as age, sex, preexisting comorbidities, and injury severity score (ISS). Of 23,705 enrolled patients, a total of 401 hypothermic patients and 13,368 normothermic patients were included in this study. Only 3.0% of patients had hypothermia upon arrival at the emergency department (ED). Compared to normothermic patients, hypothermic patients had a significantly higher rate of abbreviated injury scale (AIS) scores of ≥3 in the head/neck, thorax, and abdomen and higher ISS. The mortality rate in hypothermic patients was significantly higher than that in normothermic patients (13.5% vs. 2.3%, odds ratio (OR): 6.6, 95% confidence interval (CI): 4.86–9.01, *p* < 0.001). Of the 399 well-balanced propensity score-matched pairs, there was no significant difference in mortality (13.0% vs. 9.3%, OR: 1.5, 95% CI: 0.94–2.29, *p* = 0.115). However, multivariate logistic regression analysis revealed that patients with low body temperature were significantly associated with the mortality outcome. This study revealed that low body temperature is associated with the mortality outcome in the multivariate logistic regression analysis but not in the propensity score matching (PSM) model that compared patients with hypothermia defined as body temperatures of <36 °C to those who had normothermia. These contradicting observations indicated the limitation of the traditional definition of body temperature for the diagnosis of hypothermia. Prospective randomized control trials are needed to determine the relationship between hypothermia following trauma and the clinical outcome.

## 1. Introduction

According to the guideline for trauma patients from Advanced Trauma Life Support (ATLS), hypothermia, normothermia, and hyperthermia were defined as body temperatures of <36 °C, between 36 °C and 38 °C, and >38.1 °C, respectively, upon arrival to the emergency department (ED). In trauma patients, hypothermia, acidosis, and coagulopathy have been referred to as the lethal triad [1] that leads to a worse patient outcome. Increased mortality rates varying from 5.5% to 53.9% and even up to 100% have been reported in trauma patients with sustained hypothermia [2,3,4,5,6,7,8,9,10,11,12,13,14,15]. The etiology of hypothermia in trauma patients, including sedation, alcohol intoxication, fluid resuscitation, blood transfusion, and shock, seems to be multifactorial and interdependent [16]. Recent studies reported hypothermia to be associated with an increased injury severity [17] and a predictor of mortality in trauma patients [2,3,4,5,6,7,8,9,10,11]. However, there is controversy regarding the relationship between hypothermia and mortality outcomes, with potential confounders such as age, sex, preexisting comorbidities, shock, and injury severity. The definition of hypothermia may, however, significantly affect the observed clinical outcomes. To solve these limitations, a well-designed randomized controlled trial is needed, although it is difficult to perform in trauma patients. As an alternative, propensity score matching (PSM) is a methodology used to control for sample selection bias and to simulate the randomization process. This study aimed to assess whether hypothermia is an independent predictor of mortality in trauma patients in the condition of defining hypothermia as body temperatures of <36 °C. To achieve this study goal, the present study used a PSM model to determine the effect of hypothermia defined by ATLS on in-hospital mortality of trauma patients, and to estimate the effect of body temperature on in-hospital mortality by using multivariate logistic regression.

## 2. Methods

### 2.1. Study Design, Definitions, and Patient Selection

After receiving approval from the institutional review board of the Chang Gung Memorial Hospital (number 201700012B0), a retrospective review of the Trauma Registry System at a level I trauma center of the Kaohsiung Chang Gung Memorial Hospital [18,19] was performed to identify all adult trauma patients admitted via the ED from 1 January 2009 to 12 December 2015. The inclusion criteria consisted of patients aged at least 20 years and hospitalized for all trauma causes. The exclusion criteria included patients with missing information, such as vital signs or Glasgow Coma Scale (GCS) score; those who were dead on arrival; those who died in the ED; or those who were discharged from the ED. Patients with hyperthermia were not included. Based on their temperature upon arrival to the ED, the study population was finally divided to those displaying hypothermia (body temperatures of <36 °C) and those with normothermia (body temperatures between 36 °C and 38 °C) groups.

### 2.2. Clinical Parameters and Outcome Evaluation

Data retrieved from the two study groups included gender; sex; comorbidities; mechanisms of injury; vital signs and GCS on ED; laboratory data in the ED, which included the blood alcohol concentration (BAC), hemoglobin (Hb), hematocrit (Hct), prothrombin time (PT), and activated partial thromboplastin time (aPTT); abbreviated injury scale (AIS) score, injury severity score (ISS), hospital length of stay (LOS); rate of ICU admission; and in-hospital mortality. The primary outcome was in-hospital mortality.

### 2.3. Statistical Analysis

IBM SPSS Statistics for Windows, version 22.0 (IBM Corp., Armonk, NY, USA) was used to perform all descriptive and comparative statistics. We first compared the unadjusted variables between the hypothermia and normothermia groups. Demographic and clinical characteristics were analyzed using chi-square tests for categorical variables presented as odds ratios (ORs) with 95% confidence intervals (CIs) and analysis of variance tests for continuous variables. A multivariate logistic regression analysis was performed in order to identify factors related to mortality. Pearson correlation was used to determine if correlations were present between the biochemistry of Hb, Hct, PT, and aPTT levels and the body temperature of the trauma patients. To account for the nonrandom assignment of patients between the two groups, PSM analysis was employed to reduce the potential selection bias. The potential confounders adjusted in the PSM included age, sex, preexisting comorbidities, and ISS. After 1:1 matching with propensity scores calculated with logistic regression analysis with the aforementioned confounding factors, the patient outcomes were analyzed by conditional Cox proportional hazard regression. For this study, *p* values of <0.05 were considered to indicate statistical significance.

## 3. Results

### 3.1. Demographic Data and Clinical Characteristics of the Study Population

**Figure 1:**
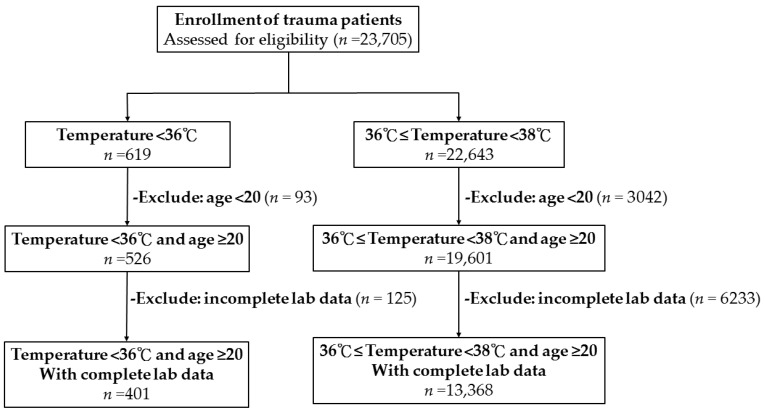
Flowchart of patient selection from the Trauma Registry System.

**Figure 2:**
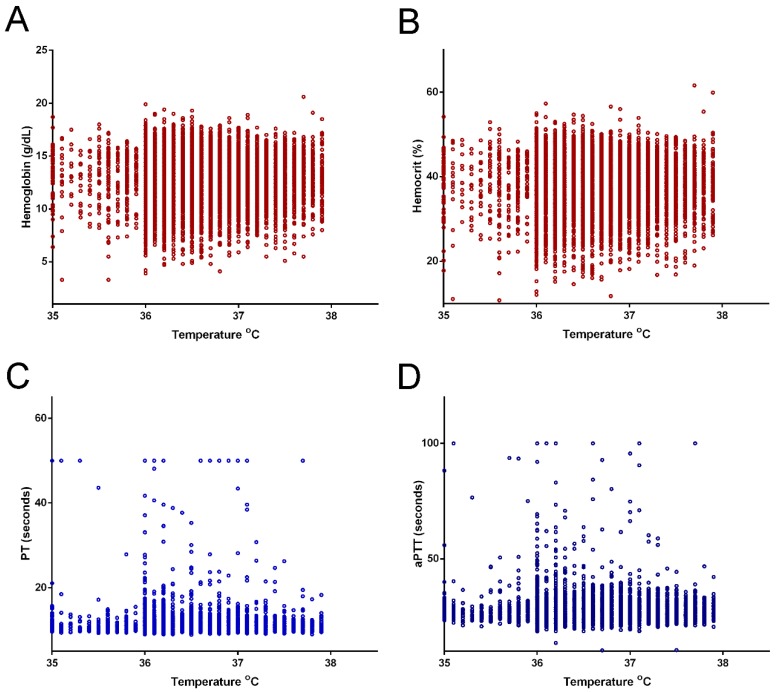
Pearson correlation of levels of hemoglobin (Hb) (**A**), hematocrit (Hct) (**B**), prothrombin time (PT) (**C**), and activated partial thromboplastin time (aPTT) (**D**) to the body temperature.

Of 23,705 enrolled patients, 401 hypothermic patients and 13,368 normothermic patients (Figure 1) were included in the analysis after filtering. Only 3.0% of patients had hypothermia upon arrival to the ED. As shown in Table 1, a significantly higher proportion of hypothermic patients were male (64.8% vs. 55.8%; *p* < 0.001). There was no significant difference in age and comorbidities between the hypothermia and normothermia groups, except that the incidence of hypertension was significantly higher in the normothermic patients. Hypothermic patients presented with a significantly higher incidence of alcohol intoxication, defined as a BAC of ≥50 mg/dL. Hypothermic patients also presented with a significantly lower systolic blood pressure (SBP) and a higher incidence of low SBP, defined either as SBP of ≤60 or 90 mmHg. There was no significant difference in heart or respiratory rates between hypothermic patients and normothermic patients. Regarding trauma mechanisms, more hypothermic patients were injured as the driver of a motor vehicle, the driver of a motorcycle, and as a victim in a strike injury, but less were injured in a fall compared to normothermic patients. Compared to normothermic patients, hypothermic patients had a significantly lower GCS score; higher rate of AIS score of ≥3 in the head/neck, thorax, and abdomen; a lower rate of AIS score of ≥3 in the extremities; and a higher ISS. The distribution of ISS according to the ISS stratification (<16, 16–24, and ≥25) revealed that more hypothermic patients had an ISS of 16–24 and ≥25 and fewer had an ISS of <16 compared to normothermic patients. The mortality rate in hypothermic patients was significantly higher than that in normothermic patients (13.5% vs. 2.3%, OR: 6.6, 95% CI: 4.86–9.01, *p* < 0.001). Hospital LOS was also significantly longer for hypothermic patients than for normothermic patients (12.4 days vs. 10.1 days, *p* = 0.001) and significantly more hypothermic patients were admitted to the ICU than were normothermic patients (43.6% vs. 21.4%, OR: 2.8, 95% CI: 2.33–3.48, *p* < 0.001). Regarding hematologic biochemistry parameters (Table 2), hypothermic patients had significantly lower hemoglobin and hematocrit levels and impaired coagulation function, as indicated by PT and aPTT, than normothermic patients (PT, 11.7 ± 4.7 vs. 10.9 ± 2.6; *p* < 0.001; aPTT, 29.6 ± 12.0 vs. 27.3 ± 4.4; *p* < 0.001). As shown in Table 3 and Figure 2, the Pearson correlation revealed that body temperature was negatively correlated with PT (correlation coefficient = −0.035, *p* < 0.001) and aPTT (correlation coefficient = −0.068, *p* < 0.001); however, these correlations were very weak. In addition, no significant correlation of body temperature to the level of Hb and Hct was found.

### 3.2. Risk Factors Associated with Mortality

As shown in Table 4, in the comparison between fatal and survival patients, the fatal patients were predominantly male, significantly older, and had higher rates of pre-existed CAD, CHF, and ESRD than those of survival patients. In addition, the fatal patients presented a significantly lower temperature, SBP, and HR, but higher ISS, than that of those survival patients. Multivariate logistic regression analysis (Table 5) revealed that patients being male, with older age, pre-existed CHF and ESRD, low body temperature, and with a higher injury severity were significantly associated with the mortality outcome.

### 3.3. Outcomes of the PSM Study Population

Propensity score-matched patients were selected to reduce the effect of differences in sex and age, preexisting comorbidities, and injury severity of the patient population on the outcome assessment. The 399 well-balanced pairs did not have significant differences in sex, age, co-morbidity, or ISS (Table 6). There were no significant differences between these two propensity score-matched study populations regarding mortality, hospital LOS, ICU admission rates, and PT and aPTT levels. Although the mortality rate remained higher in hypothermic patients, the risk of this outcome was not statistically significant (13.0% vs. 9.3%, OR: 1.5, 95% CI: 0.94–2.29, *p* = 0.115). 

## 4. Discussion

In this study, the unadjusted analysis suggested a significant association between hypothermia and mortality and multivariate logistic regression analysis revealed that patients’ low body temperature was significantly associated with the mortality outcome. However, PSM analysis in the propensity score-matched study populations with minimized potential confounding factors did not identify that hypothermia defined by the body temperatures of <36 °C was associated with increased mortality, longer hospital LOS, or ICU admission rate. These contradicting observations indicated the limitation of the traditional definition of body temperature for the diagnosis of hypothermia with a cut off at 36 °C. Without the consideration of depth of hypothermia, the mechanism that led to the hypothermia, or the rate of decline of core body temperature, the division of the trauma patients into hypothermia or normothermia according a defined body temperature is too simple for the prediction of the outcome from injury. The conflict in the observed results also reflect the current diverse opinions regarding the association of hypothermia and mortality. Most of the retrospective and prospective studies found that hypothermia is not only an ominous sign but also an independent predictor of mortality [2,3,4,5,6,7,8,9,10,11]. In contrast, Trentzsch et al. [15] analyzed 5197 trauma patients and concluded that hypothermia is just an after effect of blood loss and hemorrhagic shock and thus a result of injury severity, which is unlikely to be an independent predictor of mortality. Mommsen et al. [14] failed to prove that accidental hypothermia was an independent factor for mortality in the multivariate analysis. In addition, after correcting for injury severity, including physiologic and anatomic indicators, a retrospective study by Steinemann et al. [13] reported no difference in mortality rates between hypothermic and normothermic patients. Furthermore, a prospective observational study of trauma patients found that a body temperature of <35 °C was a significant risk factor for multiple organ dysfunction, but that early hypothermia was not an independent predictor of mortality [12].

Obviously, the differences in cut-off points for hypothermia and population characteristics would lead to diverse or even contracting observations of the outcomes. Hypothermic patients generally had higher injury severity and incidence of lower blood pressure than those in normothermic patients. Furthermore, hypothermic patients more often had severe injuries to the head, chest, and abdomen compared to normothermic patients. Therefore, it is not surprising that the vital signs of these patients were often unstable. In this study, although the injury severity had been adjusted in PSM to estimate the effect of hypothermia on mortality, it should be noted that, with the continuous variable of body temperatures being turned into a binary categorical data, the cut-off value selected for a definition of hypothermia (i.e., body temperatures of <36 °C in this study) may present a bias or even a false negative finding in the outcome measurement. For example, a much lower temperature (e.g., 32 °C or less) may even present a survival advantage for the trauma patients, as evidenced by a decreased odds ratio for death in the therapeutic hypothermia [20,21,22]. Furthermore, Reynolds et al. reported that a temperature of <34 °C in hemorrhagic patients requiring massive transfusion was associated with a two-fold increased risk of mortality [11]. Klauke et al. and Waibel et al. defined hypothermia as a core body temperature of <36 °C [3,5], whereas other studies defined hypothermia as a core body temperature of <35 °C [6,8]. Our current study observed hypothermia, defined as a temperature of <36 °C, in 3.0% of patients, with a 13.5% mortality rate for hypothermic patients. Our incidence was less than the previously reported incidences of 15.7–22.9% in which hypothermia was similarly defined as <36 °C [3,5]. The lower incidence of hypothermia may be attributed to the location of our institution in southern Taiwan, which has a subtropical climate with less chance of hypothermia exposure [23,24], as well as the convenient access that decreases the pre-hospital time to care. However, the mortality rate of hypothermic patients in this study was similar to that reported in previous studies, which ranged from 7.4% to 14.6% [3,5]. 

Hypothermia-induced coagulopathy is of potential physiological concern. Although coagulopathy is a member of the lethal triad [1], significant coagulopathy does not develop until below 34 °C. Moreover, hypothermia coagulopathy occurs even at a high core temperature and reversible platelet dysfunction occurs during hypothermia, which is the probable source of dysfunctional bleeding noted with hypothermia [25]. A study on the impact of hypothermia on platelet function in a porcine model of multiple trauma reported that mild hypothermia affects the coagulation system but does not aggravate trauma-induced coagulopathy [26]. In this study, the Pearson correlation revealed that the body temperature was negatively correlated with PT and aPTT; however, these correlations are very weak. The propensity score-matched hypothermic patients also did not present significant coagulopathy compared to normothermic patients. However, this observation may also be limited by the traditional definition of body temperature for the diagnosis of hypothermia. 

Although the incidence of hypothermia at the time of admission varies between 1.57% and 44.6% in trauma patients [2,3,4,5,6,7,8,9,10,11,12,13,14,15], attending physicians may fail to identify hypothermia in the ED. In a retrospective analysis of the association between hypothermia on admission and clinical outcomes in 58,947 major trauma patients, 8.8% of patients were excluded due to temperature not being recorded [7]. In a retrospective analysis of 642 trauma admissions in a trauma center, up to 33% of patients had no temperature recorded in the ED [27]. The effect of hypothermia may even be overlooked in regions with tropical or subtropical climates, such as in Taiwan, where the risk of low temperature exposure is relatively low. This study presented, with contrasting results, an unsolved problem regarding the impact of admission body temperature on the trauma patients. Prospective randomized control trials are needed to determine the relationship between hypothermia following trauma and the clinical outcome.

This study had several other limitations. First, the retrospective design and the use of data retrieved from a single center resulted in an inherent bias. Second, potential confounders (e.g., infused fluid volume and temperature, blood transfusion, ventilator use, time interval from the initial scene to the ED, and resuscitation during the transportation to the ED) were not controlled for in this study and may have led to a selection bias. Finally, patients declared dead at the scene of an accident or upon hospital arrival were not included in the database, which may have led to a selection bias.

## 5. Conclusions

This study revealed that low body temperature was associated with the mortality outcome in the multivariate logistic regression analysis but not in the PSM model that compared patients with hypothermia defined as body temperatures of <36 °C to those who had normothermia. These contradicting observations indicated that there is limitation for the diagnosis of hypothermia according to the traditional definition of body temperature. Prospective randomized control trials are needed to determine the relationship between hypothermia following trauma and the clinical outcome.

## Figures and Tables

**Table 1:** Demographics and admission characteristics of the study groups.

Variables	Hypothermia *n* = 401		Normothermia *n* = 13,368		Odds Ratio (95% CI)		*p*
Sex, *n* (%)							<0.001
Male	260	(64.8)	7455	(55.8)	1.5	(1.19–1.80)	
Female	141	(35.2)	5913	(44.2)	0.7	(0.56–0.84)	
Age (years)	53.3	±19.0	54.3	±19.4	—		0.293
Comorbidity, *n* (%)							
HTN	99	(24.7)	3972	(29.7)	0.8	(0.62–0.98)	0.030
DM	59	(14.7)	2119	(15.9)	0.9	(0.69–1.21)	0.579
CVA	13	(3.2)	589	(4.4)	0.7	(0.42–1.27)	0.270
CAD	16	(4.0)	554	(4.1)	1.0	(0.58–1.60)	0.901
CHF	5	(1.2)	125	(0.9)	1.3	(0.54–3.29)	0.594
ESRD	11	(2.7)	288	(2.2)	1.3	(0.70–2.36)	0.484
BAC ≥ 50 mg/dL, *n* (%)	60	(15.0)	871	(6.5)	2.5	(1.90–3.35)	<0.001
Alcohol level (mg/dL)	213.0	±90.4	187.1	±73.3	—		0.010
SBP (mmHg)	137.8	±46.0	151.4	±34.2	—		<0.001
SBP ≤ 60 mmHg, *n* (%)	18	(4.5)	45	(0.3)	13.9	(7.98–24.26)	<0.001
SBP ≤ 90 mmHg, *n* (%)	46	(11.5)	307	(2.3)	5.5	(3.97–7.65)	<0.001
HR (beats/min)	83.9	±26.4	86.3	±17.4	—		0.109
RR (times/min)	18.7	±5.0	18.6	±2.0	—		0.741
Mechanism, *n* (%)							
Driver of MV	16	(4.0)	224	(1.7)	2.4	(1.45–4.09)	0.002
Passenger of MV	5	(1.2)	97	(0.7)	1.7	(0.70–4.27)	0.225
Driver of motorcycle	195	(48.6)	5380	(40.2)	1.4	(1.15–1.72)	<0.001
Passenger of motorcycle	4	(1.0)	270	(2.0)	0.5	(0.18–1.32)	0.200
Bicyclist	18	(4.5)	506	(3.8)	1.2	(0.74–1.93)	0.506
Pedestrian	7	(1.7)	277	(2.1)	0.8	(0.39–1.79)	0.727
Fall	96	(23.9)	4611	(34.5)	0.6	(0.47–0.75)	<0.001
Penetrating injury	11	(2.7)	563	(4.2)	0.6	(0.35–1.18)	0.163
Blunt injury	28	(7.0)	1168	(8.7)	0.8	(0.53–1.16)	0.242
Strike by/against	21	(5.2)	272	(2.0)	2.7	(1.69–4.20)	<0.001
GCS	12.2	±4.5	14.2	±2.3	—		<0.001
≤8	89	(22.2)	688	(5.1)	5.3	(4.10–6.74)	<0.001
9–12	29	(7.2)	551	(4.7)	1.8	(1.23–2.67)	0.003
≥13	283	(70.6)	12,129	(90.1)	0.2	(0.20–0.31)	<0.001
AIS ≥ 3							
Head/neck	154	(38.4)	2728	(20.4)	2.4	(1.98–2.99)	<0.001
Face	1	(0.2)	33	(0.2)	1.0	(0.14–7.41)	1.000
Thorax	50	(12.5)	855	(6.4)	2.1	(1.54–2.83)	<0.001
Abdomen	18	(4.5)	339	(2.5)	1.8	(1.11–2.93)	0.019
Extremity	105	(26.2)	4321	(32.3)	0.7	(0.59–0.93)	0.011
ISS (median, IQR)	10	(5–20)	9	(4–10)	—		<0.001
<16	236	(58.9)	10,929	(81.8)	0.3	(0.26–0.39)	<0.001
16–24	86	(21.4)	1712	(12.8)	1.9	(1.46–2.37)	<0.001
≥25	79	(19.7)	727	(5.4)	4.3	(3.30–5.52)	<0.001
Mortality, *n* (%)	54	(13.5)	307	(2.3)	6.6	(4.86–9.01)	<0.001
Hospital LOS (days)	12.4	±12.9	10.1	±10.8	—		0.001
ICU admission, *n* (%)	175	(43.6)	2858	(21.4)	2.8	(2.33–3.48)	<0.001

Data are presented as means ± SD, median (IQR), or *n* (%). HTN, hypertension; DM, diabetic mellitus; CVA, cerebral vascular accident; CAD, coronary artery disease; CHF, congestive heart failure; ESRD, end-stage renal disease; BAC, blood alcohol concentration; SBP, systolic blood pressure; HR, heart rate; RR, respiratory rate; MV, motor vehicle; GCS, Glasgow coma score; AIS, abbreviated injury scale; ISS, injury severity scale; LOS, length of stay; ICU, intensive care unit.

**Table 2:** Comparison of biochemistry between study groups.

Variables	Hypothermia *n* = 401		Normothermia *n* = 13,368		Odds Ratio (95% CI)	*p*
Hemoglobin (g/dL)	12.8	±2.5	13.1	±2.1	—	0.016
Hematocrit (%)	38.0	±6.9	38.8	±6.4	—	0.022
PT (seconds)	11.7	±4.7	10.9	±2.6	—	<0.001
aPTT (seconds)	29.6	±12.0	27.3	±4.4	—	<0.001

Data are presented as means ± SD. PT, prothrombin time; aPTT, activated partial thromboplastic time.

**Table 3:** Correlation of biochemistry to temperature in study population.

Variables	Pearson Correlation	*p*
Hemoglobin (g/dL)	−0.011	0.067
Hematocrit (%)	−0.016	0.067
PT (seconds)	−0.035	<0.001
aPTT (seconds)	−0.068	<0.001

PT, prothrombin time; aPTT, activated partial thromboplastic time.

**Table 4:** Comparison of variable between fatal and survival patient groups.

Variables	Mortality *n* = 361		Survival *n* = 13,408		*p*
Gender, *n* (%)					<0.001
Male	241	(66.8)	7474	(55.7)	
Female	120	(33.2)	5934	(44.3)	
Age (years)	60.4	±19.7	54.1	±19.3	<0.001
Co-morbidity, *n* (%)					
HTN	118	(32.7)	3953	(29.5)	0.198
DM	61	(16.9)	2117	(15.8)	0.609
CVA	22	(6.1)	580	(4.3)	0.116
CAD	26	(7.2)	544	(4.1)	0.005
CHF	8	(2.2)	122	(0.9)	0.021
ESRD	22	(6.1)	277	(2.1)	<0.001
Temperature (°C)	36.0	±2.8	36.5	±0.6	0.002
SBP (mmHg)	144.6	±57.0	151.2	±33.8	0.029
HR (beats/min)	47.8	±35.0	65.5	±29.4	<0.001
RR (times/min)	18.5	±6.0	18.6	±1.9	0.661
ISS (median, IQR)	25	(17–30)	9	(4–10)	<0.001

Data are presented as means ± SD, median (IQR), or *n* (%). HTN, hypertension; DM, diabetic mellitus; CVA, cerebral vascular accident; CAD, coronary artery disease; CHF, congestive heart failure; ESRD, end-stage renal disease; SBP, systolic blood pressure; HR, heart rate; RR, respiratory rate; ISS, injury severity scale.

**Table 5:** Variables related to mortality of the patients in multivariate logistic regression.

Variables	Odds Ratio	(95% CI)	*p*
Male	1.72	1.32–2.24	<0.001
Age	1.04	1.03–1.04	<0.001
CHF	2.71	1.24–5.92	0.012
ESRD	3.69	2.23–6.11	<0.001
Temperature (°C)	0.66	0.54–0.80	0.002
HR (beats/min)	0.99	0.99–0.99	<0.001
ISS (median, IQR)	1.17	1.16–1.19	<0.001

CHF, congestive heart failure; ESRD, end-stage renal disease; HR, heart rate; ISS, injury severity scale.

**Table 6:** Comparison of outcomes between study groups before and after propensity score matching.

Varibles	Before							After						
	Hypothermia *n* = 401		Normothermia *n* = 13,368		*OR (95% CI)*		*p*	Hypothermia *n* = 399		Normothermia *n* = 399		*OR (95% CI)*		*p*
Sex, *n* (%)														1.000
Male	260	(64.8)	7455	(55.8)	1.5	(1.19–1.80)	*<0.001*	258	(64.7)	258	(64.7)	1.0	(0.75–1.34)	
Female	141	(35.2)	5913	(44.2)	0.7	(0.56–0.84)	*<0.001*	141	(35.3)	141	(35.3)	1.0	(0.75–1.34)	
Age (years)	53.3	±19.0	54.3	±19.4	—		*0.293*	53.2	±19.0	53.1	±19.2	—		0.969
Comorbidity, *n* (%)														
HTN	99	(24.7)	3972	(29.7)	0.8	(0.62–0.98)	*0.030*	98	(24.6)	98	(24.6)	1.0	(0.72–1.38)	1.000
DM	59	(14.7)	2119	(15.9)	0.9	(0.69–1.21)	*0.579*	57	(14.3)	57	(14.3)	1.0	(0.67–1.49)	1.000
CVA	13	(3.2)	589	(4.4)	0.7	(0.42–1.27)	*0.270*	13	(3.3)	13	(3.3)	1.0	(0.46–2.19)	1.000
CAD	16	(4.0)	554	(4.1)	1.0	(0.58–1.60)	*0.901*	15	(3.8)	15	(3.8)	1.0	(0.48–2.07)	1.000
CHF	5	(1.2)	125	(0.9)	1.3	(0.54–3.29)	*0.594*	5	(1.3)	5	(1.3)	1.0	(0.29–3.48)	1.000
ESRD	11	(2.7)	288	(2.2)	1.3	(0.70–2.36)	*0.484*	11	(2.8)	11	(2.8)	1.0	(0.43–2.33)	1.000
ISS (median, IQR)	10	(5–20)	9	(4–10)	—		*<0.001*	10	(5–20)	10	(5–20)	—		0.950
Mortality, *n* (%)	54	(13.5)	307	(2.3)	6.6	(4.86–9.01)	*<0.001*	52	(13.0)	37	(9.3)	1.5	(0.94–2.29)	0.115
LOS in hospital (days)	12.4	±12.9	10.1	±10.8	—		*0.001*	12.4	±12.9	12.8	±13.7	—		0.662
ICU admission, *n* (%)	175	(43.6)	2858	(21.4)	2.8	(2.33–3.48)	*<0.001*	173	(43.4)	159	(39.8)	1.2	(0.87–1.53)	0.350
PT (seconds)	11.7	±4.7	10.9	±2.6	—		*<0.001*	11.2	±2.9	11.3	±9.6	—		0.812
aPTT (seconds)	29.6	±12.0	27.3	±4.4	—		*<0.001*	28.3	±9.6	27.6	±5.0	—		0.145

Data are presented as means ± SD, median (IQR), or *n* (%). HTN, hypertension; DM, diabetic mellitus; CVA, cerebral vascular accident; CAD, coronary artery disease; CHF, congestive heart failure; ESRD, end-stage renal disease; ISS, injury severity scale; LOS, length of stay; ICU, intensive care unit; PT, prothrombin time; aPTT, activated partial thromboplastic time.
